# miR-378d suppresses malignant phenotype of ESCC cells through AKT signaling

**DOI:** 10.1186/s12935-021-02403-y

**Published:** 2021-12-22

**Authors:** Jie Peng, Susu Shi, Juan Yu, Jianli Liu, Haixiang Wei, Haixia Song, Shaoqiang Wang, Zhejie Li, Shujin He, Lei Li, Hongyan Zhang, Zhizhen Yan, Ran Zhao, Yukun Liu, Yanrong Liu, Junjun Li, Renya Zhang, Wei Wang

**Affiliations:** 1 Department of Pathology, Affiliated Hospital of Jining Medical University, Jining Medical University, 89 Guhuai Road, Jining, 272029 Shandong China; 2 Department of Pathology, Zibo Central Hospital, Zibo, 255036 Shandong China; 3 Department of Pathology, The First People’s Hospital of Xuzhou, Xuzhou, 221106 Jiangsu China; 4 Department of Thoracic Surgery, Affiliated Hospital of Jining Medical University, Jining Medical University, Jining, 272029 Shandong China; 5 Cancer Research Institute and School of Basic Medical Sciences, Central South University, Changsha, 410078 Hunan China

**Keywords:** miR-378d, ESCC, AKT1, Chemo-resistance, Prognosis

## Abstract

**Background:**

Post-resistance progress in paclitaxel (PTX) treatment remains a major challenge in tumor treatment. A high dose of PTX was used for cell lines to analyze the changes in molecular expression. The miR-378d was sharply reduced in surviving cells, but the role of miR-378d in Esophageal squamous cell carcinoma (ESCC) remained unclear.

**Methods:**

We analyzed the relationship between miR-378d expression and the clinicopathological features of ESCC. We constructed miR-378d silent expression cell lines to study phenotypes and molecular mechanisms.

**Results:**

The miR-378d expression was associated with good prognosis in patients with ESCC. miR-378d inhibition promoted chemo-resistance, monoclonal formation, EMT, migration, invasion, stemness, and metastasis of ESCC cells. miR-378d can target downregulated AKT1.

**Conclusions:**

Therefore, miR-378d expression is a good prognostic factor of patients with ESCC and regulates the malignant phenotype of tumor cells through AKT.

**Supplementary Information:**

The online version contains supplementary material available at 10.1186/s12935-021-02403-y.

## Background

Great progress has been made in ESCC diagnosis and treatment in recent years, but the five-year survival rate of patients with ESCC remains low, and no effective targeted therapy is available [1]. Chemotherapy, adjuvant therapy, and radiotherapy are still the main treatments for ESCC, particularly for patients in advanced stages. Acquired chemoresistance is a frequent cause of treatment failure; it leads to local recurrence and metastasis [2, 3].

Paclitaxel (PTX) is a first-line chemotherapy drug for ESCCs and can stabilize microtubule polymers to block cell cycle during the G2-M phase, thus hindering the development of mitosis and forming PGCCs [4, 5]. Studies reported that AKT promotes PTX resistance in multiple tumors. IL-22 enhanced the paclitaxel resistance of lung adenocarcinoma cells by promoting the expression of AKT and Bcl-2 [6]. Calpain-2 promotes NSCLC progression and contributes to paclitaxel resistance by activating the EGFR/pAKT pathway [7]. Inhibition of AKT by specific PI3K-AKT inhibitors (Wortmannin and LY294002) synergistically increased the efficacy of the paclitaxel-induced apoptosis in ovarian cancer [8]. Approximately 15.7% of AKT1 amplification is found in ESCC [9], and the AKT signaling pathway plays an important role in ESCC metastasis [10]. Xanthohumol significantly inhibits AKT kinase activity in an ATP-competitive manner and decreases tumor volume and weight in patient-derived xenografts (PDXs) highly expressing AKT. However, xanthohumol has no effect on PDXs lowly expressing AKT in vivo [11]. AKT is also involved in cellular polyploidization; AKT overexpression restored polyploidy in death effector domain-containing protein (DEDD) deficient mouse decidual cells [12]. Yap strongly induced acetyltransferase p300-mediated E3 ligase acetylation through AKT signaling; the Skp2 of acetylation is limited to the cytosol, leading to excessive accumulation of cyclin-dependent kinase inhibitor p27 and resulting in the cessation of mitosis and subsequent cellular polyploidy [13].

microRNAs (miRNAs) play an important role in tumor chemotherapeutic resistance and progression [14–16] and can be used to regulate PCC formation by their target genes. In the present study, we induced chemo-resistant cells by using PTX, and their miRNA expression profiles were analyzed and compared with those of normal cultured cancer cells. The miR-378d expression was significantly downregulated in surviving cells through PTX treatment. However, the role of miR-378d in ESCC remains unclear.

## Materials and methods

### Cell culture

Four human ESCC cell lines (KYSE-30, KYSE-150, KYSE-510, and TE-1) were obtained from the German Resource Center for Biological Material (DSMZ), and 293 T cells were purchased from the American Type Culture Collection (USA). The ESCC cell lines were cultured in RPMI1640 (293 T cells in DMEM) supplemented with 10% fetal bovine serum (FBS; (#04-001-1ACS, BI), 100 units/mL of penicillin, and 100 μg/mL of streptomycin and maintained at 37 °C and 5% CO_2_.

### ESCC organization source

A cohort of 610 subjects with ulcerative ESCCs was recruited between 2008 and 2014 from the Department of Thoracic Surgery, the Affiliated Hospital of Jining Medical University (Shandong, PR China). We collected relevant clinical data and prognostic information of patients. A total of 470 cases were male, and 140 cases were female (3.4:1) with ages in the range of 34–83 years old (mean age = 61 years old). A total of 318 patients had long-term follow-up results, and the mean survival time was 29 months (1–95.2 months).

All biopsies were immediately fixed in 4% buffered paraformaldehyde, routinely processed, and embedded with paraffin. Tumors were classified according to the standard TNM staging guidelines of UICC (TNM Classification of Malignant Tumours, Eighth edition). The study protocol was reviewed and approved by the local ethics committee. All patients provided written consent for the use of their tissue samples. This research was approved by the ethics committee of Jining Medical University. Each patient signed an informed consent form. This study was approved by the institutional review board of the Affiliated Hospital of Jining Medical University, Jining, China (2018-FY-040).

### Tissue microarray

Representative areas of the ESCC were marked on each slide stained with hematoxylin–eosin (H&E) and tissue paraffin block. The marked areas of tissue paraffin blocks were sampled for TMAs. TMAs were assembled with a tissue-arraying instrument (Beecher Instruments, Silver Springs, MD, USA), as described by Kallioniemi et al*.* [17].

### In situ hybridization

ESCC TMA was dewaxed in xylene, rehydrated in alcohol gradient, and washed twice with DEPC-PBS. The sections were treated with 2 μg/mL proteinase K (Roche) for 15 min at 37 °C and washed thrice with DEPC-PBS. Then, the sections were acetylated for 15 min at room temperature (acetic anhydride in DEPC-water, 6 N HCl, and triethanolamine) and subsequently washed thrice with DEPC-PBS. Sections were prehybridized in hybridization buffer (50% formamide; 5 × saline sodium citrate; pH 7.0; 100 μg/mL sheared salmon sperm DNA, 0.5 mg/mL yeast tRNA, and 1 × Denhardt’s solution) at 58 °C for 1 h before the buffer was replaced with hybridization solution containing the miR probe. The miR-378d detection probes labeled with digoxin at 5′-end were from Boster. Probes were diluted in pre-hybridization buffer to achieve a concentration of 5 nM and hybridized with the sections overnight at 58 °C according to the RNA melting temperature of probes. After hybridization, the sections were washed thrice with 2 × SSC and 0.2 × SSC, permeabilized for immunostaining with 0.1% Triton X-100, and washed twice with PBS. Unspecific background was blocked with 5% swine serum diluted in PBS/BSA for 30 min.

### PTX treatment

All cell lines were cultured in complete medium until the cells reached 90% confluence. PTX at different concentrations was added to the different cells, which were then treated for 24 h. PTX was then withdrawn, the medium was replaced, and the cells were cultured until no significant cell death was observed.

### Sequencing of miRNA and microarray analysis

The small RNAs of TE-1 control and TE-1-PTX (9 days) treated with PTX were used for miRNA sequencing. The miRNA-sequencing libraries were constructed according to the protocol for the Illumina small RNA sample preparation kit. Sequencing was performed on an Illumina HiSeq 2000 sequencer. Library construction and sequencing were performed at Genergy Biotech (Shanghai). miRNA expression was analyzed with miRdeep 2.0.0.7 [18], and differentially expressed miRNAs were identified using an FDR cutoff value of 0.05. mRNA expression profiling was conducted by using Roche NimbleGen Human 12 × 135 K Gene Expression Array by KangChen Bio-tech. Raw data were processed with RMA algorithm, and differential expression analysis was performed with R package limma37 (Version 3.22.7).

### Cell transfection

Plasmid transfection was performed using Lipofectamine™ 3000 reagent (Invitrogen, USA) according to the manufacturer’s instructions. Transfection of miR-NC, miR-378d-mimics, or miR-378d-inhibitor (Ribobio, China) was performed using Lipofectamine RNAiMAX (Invitrogen, USA) at a final concentration of 20 nM.

### Lentivirus packaging and transduction

Vectors were packaged in 293FT cells using ViraPower Mix (Genepharma). After culturing for 48 h, lentiviral particles in the supernatant were harvested and filtered by centrifugation at 500*g* for 10 min and transfected into the ESCC cells. The cells were then cultured under puromycin (10 μg/mL) selection for 2 weeks, after which real-time PCR was used to determine the level of miR-378d. Cell lines stably expressing miR-378d-inhibitor or negative control (NC) vector were designated as Lv-miR-378d-inhibitor and Lv-miR-NC cells, respectively.

### Western blot

Cells were lysed in ice-cold RIPA buffer containing a protease-inhibitor cocktail (Roche). Protein content was quantified with a BCA protein assay kit (Thermo Fisher Scientific). Approximately 30 μg of protein was subjected to electrophoresis, transferred onto PVDF membranes (Millipore), and blocked with 5% nonfat dry milk in Tris-buffered saline containing 0.1% Tween 20. Membranes were incubated overnight with the following primary antibodies: anti-AKT (dilution 1:1000; CST), anti-AKT1 (1:1000; CST), anti-p-AKT(Ser473) (1:1000; CST), anti-p-AKT (Thr308) (1:1000; CST), anti-β-catenin (1:2000; Proteintech), anti-ALDH1A1 (1:1000; Abcam), anti-Vimentin (1:500; CST), anti-RhoA antibody (1:500; Proteintech), anti-PARP antibody (1:1000; CST), anti-GAPDH antibody (1:3000; Proteintech), and anti-α-tubulin antibody (1:3000; Proteintech), which served as endogenous controls. The specific bands were visualized using secondary anti-rabbit or anti-mouse antibody (1:3000; Proteintech), enhanced chemiluminescence detection kit (Millipore), and FluorChem FC2 Multi-Imager II (Alpha Innotech).

### Transwell migration and invasion assay

In vitro cell migration assay was performed using transwell chambers (8 μm pore size; Corning). Cells were plated in serum-free medium (2 × 10^4^ cells per transwell). Medium containing 15% FBS in the lower chamber served as the chemoattractant. After 48 h, the nonmigrating cells were removed from the top face of the filters by using cotton swabs. The migratory cells located on the bottom side of the chamber were stained with crystal violet, air dried, photographed, and counted. Images of five random fields at 10× magnification were captured from each membrane, and the number of migratory cells was counted. Similar inserts coated with Matrigel were used to determine the cellular invasive potential in the invasion assay.

### Matrigel 3D cell culture

Cells (5 × 10^3^/50 μL) were seeded onto 96-well plates with a round-bottom lid made of ultralow attachment polystyrene (#7007, Costar, USA). The cells were cultured overnight and formed one sphere per well. After discarding the medium and adding 75 μL of melted Matrigel (BD, USA) to resuspend the cell sphere, the mixture was incubated for 30 min for settling. Finally, 200 μL of full medium/well was added, and the medium was changed every other day.

### 3D culture scaffolds

For 3D cultures, the commercial 200 μm-thick scaffolds (Alvetex^®^, ReproCELL, Durham, UK) were used and manipulated according to the manufacturer’s instructions. To make the scaffold hydrophilic, inserts were first submerged in 70% ethanol for 1 min. Then, they were washed twice with sterile PBS and once with 10% FBS RPMI-1640 complete medium. In each experiment, 2 × 10^6^ cells were seeded in either Alvetex^®^ scaffold placed in a 12-well plate (covered by 3.5 mL of complete medium). Cultured for 7 days.

### Colony formation assays

Cells were seeded onto six-well plates (1 or 10 × 10^2^ cells per plate) and cultured for 10 days. The colonies were stained with 1% crystal violet for 30 s after fixation with 10% formaldehyde for 15 min and then imaged using the camera of an iPhone 5S (Apple, Inc., Cupertino, CA, USA).

### Dual-luciferase reporter assay

In a typical procedure, 293 T cells (3 × 10^4^ cells per well) grown on a 24-well plate were co-transfected with luciferase reporter miRGLO-AKT1-3′UTR plasmid (WT or mutation type; Genepharma, Shanghai, China) (200 ng per well) and miR-378d mimics (20 nM) using Lipofectamine™ 3000 (Invitrogen, USA). Approximately 24 h later, a dual-luciferase reporter assay kit (Promega, USA) was used to measure the luciferase and *Renilla* activity of these samples according to the manufacturer’s instructions.

### F-actin cytoskeleton fluorescence staining

Cells were grown on laminin-coated glass cover slips, fixed in 4% paraformaldehyde, stained with Phalloidin (Molecular Probes, USA), and observed using a fluorescence microscope (Leica, Germany).

### Cell-viability assay

CCK8 was used to assess cell viability. KYSE510 and TE-1 cells (1 × 10^4^) were seeded onto a 96-well plate in quintuplicate per well. Approximately 12 h later, the cells were incubated with a gradient concentration of therapeutic drugs for 48 h. The medium was removed. RPMI1640 (90 μL) and CCK8 (10 μL) were subsequently added to each well, and the mixture was incubated for 3 h at 37 °C. A microplate reader was used to measure the optical density (OD) at 450 nm. The degree of drug response for tumor cells was estimated by dividing the half-maximal inhibitory concentration (IC50).

### Immunohistochemistry (IHC) and Immunofluorescence (IF)

IHC analysis was performed on the cell-block sections from the cultured cells by using the following primary antibodies: anti-CD133 antibody (1:100; Proteintech), anti-CD44 (1:100, Proteintech), anti-c-MYC (1:100; Proteintech), and anti-Ki-67 (MXB, Fuzhou, China).

Immunofluorescence and confocal microscopy were performed as previously reported [19, 20]. Images were captured by using a Zeiss confocal microscope.

### Sphere-formation assay

Sphere formation assay was performed as previously reported [20, 21]. The KYSE-150 and TE-1 cells were seeded in low-attachment six-well culture plates (Corning, NY, USA) at a density of 1 × 10^4^ cells per well under serum-free conditions consisting of DMEM/F-12 (Life Technologies), 20 ng/mL of epidermal growth factor (Invitrogen), 20 ng/mL of basic fibroblast growth factor (Invitrogen), and 20 μL/mL of B27 (Life Technologies). Images were captured under a microscope after 14 days, and the numbers of spheres (diameter ≥ 100 μm) in all wells were counted.

### Liver transplantation

The animal protocol was approved by the ethical review committee of the Affiliated Hospital of Jining Medical University. Eight-week-old male BALB/c nude mice (Beijing Weitong Lihua Laboratory Animal Technology Co., Ltd.) were anesthetized with 4% chloraldehyde hydrate (100 μL/10 g), and the body temperature was maintained by using heating blankets. The thoracoabdominal skin of nude mice was sterilized with 75% alcohol and iodophor. After cutting open the skin at the lower right of the cartilage to expose the lobe of the liver, 50 μL of cell suspension (25 μL of serum-free and 25 μL of Matrigel containing 5 × 10^5^ cells) was injected into the liver capsule slowly. Then, the syringe was pulled out, and the injection port was pressed for 2–5 min with an iodophor cotton ball. Finally, the incision was sutured layer by layer according to the anatomical structure. Mice were sacrificed 44 days after tumor cell inoculation. Afterward, liver tissues, lung tissues, and abdominal metastasis tumors were fixed in 4% saline-buffered formalin, embedded in paraffin, sectioned at 4 μm, and stained with H&E and IHC.

### Statistical analysis

Statistical analyses were performed using the SPSS 13.0 software package (SPSS, Chicago, IL, USA) and GraphPad Prism Software (version 6, La Jolla, CA, USA). For the statistical comparison of two groups, two-sided Student’s *t* test with the same variances was used. Differences between variables were analyzed by two-tailed or Fisher exact tests. Survival curves were plotted using the Kaplan–Meier method and compared with log-rank tests. Multivariate survival analysis was performed for all parameters that were significant in the univariate analysis using a Cox regression model. Comparisons between groups for statistical significance were performed with a two-tailed Student *t* test. Data were presented as the mean ± SD. P values < 0.05 were considered significant.

## Results

### Patients with a high expression of miR-378d had good prognosis

**Fig. 1:**
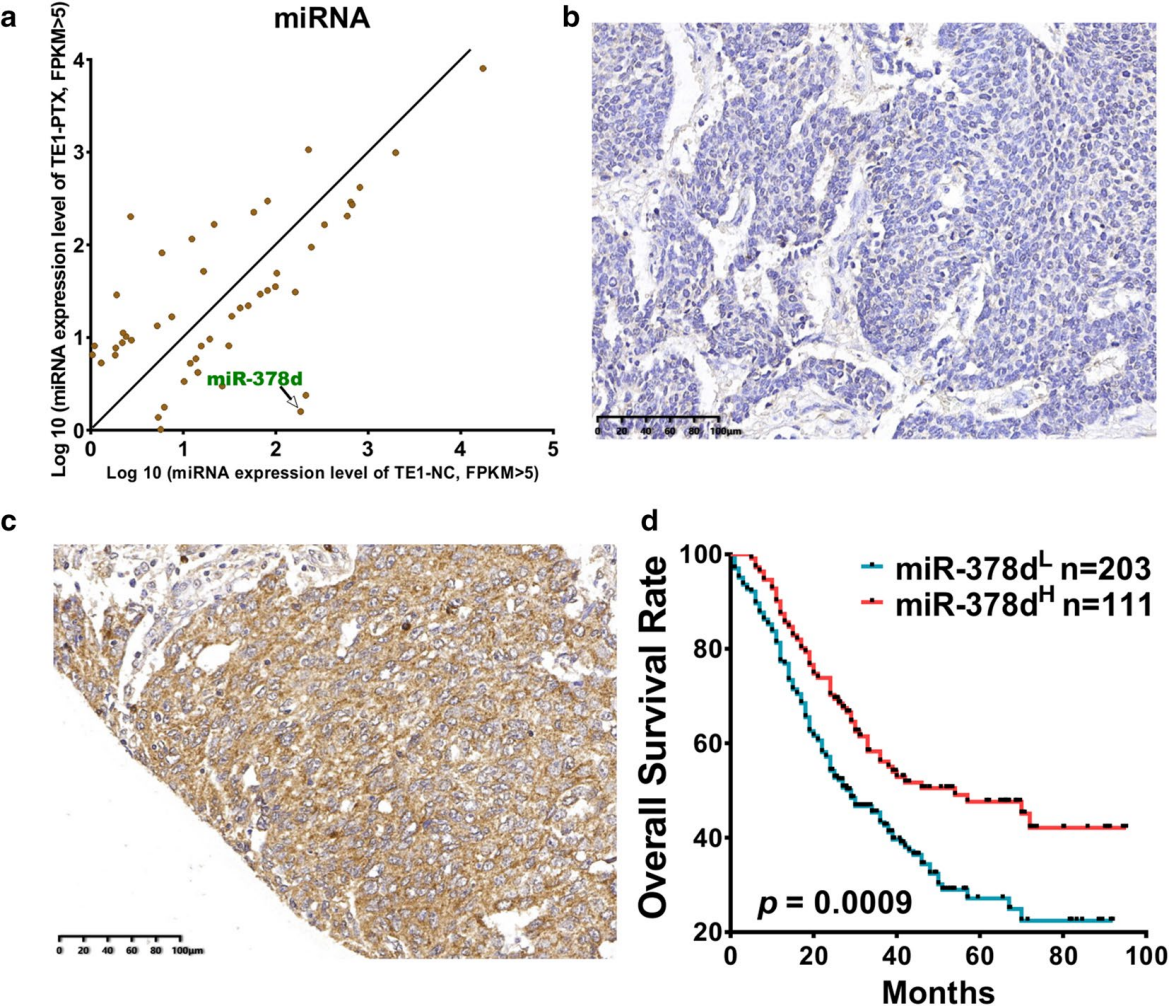
Patients with low miR-378d expression had poor prognosis**. a** Scatterplot showing differential mi-RNA expression between TE-1 Ctrl and TE-1 PTX (9 days), FPKM > 5, miR-378d expression level was significantly reduction in PTX-treated cells. **b**, **c** miR-378d expression was detected by in situ hybridization using the ESCC TMA. **b** Low expression of miR-378d. **c** high expression of miR-378d. **d** Overall survival rate of patients with ESCC according to miR-378d expression levels

PTX is the first-line drug for ESCC. In this study, we used PTX at a high concentration (300 nM) to treat TE-1 cells. From days 2 to 9, the cells died continuously, and only a small number of cells remained at day 9. These surviving cells slowly proliferated, and no more death occurred. The remaining PTX-treated cells at day 9 (TE-1-PTX) and DMSO-treated cells (TE-1-NC) were used to detect miRNA differential expression by sequencing. miRNA gene-expression profile data showed that miR-378d was significantly downregulated in the remaining cells (Fig. 1a). miR-378d has two different chromosomes in humans named MIR378D1 and MIR378D2. The mature miRNA sequence is the same. The CCLE data (https://depmap.org/portal/gene) showed that MIR378D1 was not expressed in all cell lines (Additional file 1: Fig. S1a), whereas MIR378D2 was expressed in most cell lines and ranked the second highest in esophageal cell lines (Additional file 1: Fig. S1b), thereby showing that the miR-378d is MIR378D2.

Then, we detected miR-378d expression by ISH in an ESCC tissue array containing 610 ESCC samples, because the incompleteness of the paraffin section and antigen repair led to tissue shedding. Finally, 596 specimens were obtained. Most patients with ESCC (388/596, 65.10%) showed a low expression of miR-378d (Fig. 1b). miR-378d positive staining (208/596, 34.90%) was primarily found in the cytoplasm of squamous epithelium (Fig. 1c). The miR-378d expression level had no clinicopathological significance with age (*P* = 0.269), gender (*P* = 0.864), tumor size (*P* = 0.757), differentiation (*P* = 0.249), stage (*P* = 0.305), lymphatic metastasis (*P* = 0.296), nerve invasion (*P* = 0.141), vascular invasion (*P* = 0.578), and invasion depth (*P* = 0.101) of patients with ESCC (Table 1). However, our data showed that miR-378d expression was significantly negatively associated with the overall survival rate (Fig. 1d; *P* = 0.0009) of patients with ESCC.

**Table 1:** miR-378d expression in ESCC patients and its clinicpathological significance, 596 case

Clinical information	miR-378d^Low^	miR-378d^High^	total	χ^2^	*P*
	N (%)	N (%)			
Age(years)					
≥ 61	198(63.06)	116(36.94)	314	1.220	0.269
< 61	190(67.38)	92(32.62)	282		
Gender					
Male	299(65.28)	159(34.72)	458	0.029	0.864
Female	89(64.49)	49(35.08)	138		
Tumor size(cm)					
> 4	143(65.90)	74(34.10)	217	0.096	0.757
≤ 4	245(64.64)	134(35.36)	379		
Stage					
I + II	112(68.29)	52(31.71)	164	1.052	0.305
III + IV	275(63.81)	156(36.19)	431		
LNM					
Negative	199(63.17)	116(36.83)	315	1.091	0.296
Positive	189(67.26)	92(32.74)	281		
Nerve invasion					
Negative	343(66.22)	175(33.78)	518	2.168	0.141
Positive	45(57.69)	33(42.31)	78		
Vascular invasion					
Negative	359(64.80)	195(35.20)	554	0.310	0.578
Positive	29(69.05)	13(30.95)	42		
Differentiation					
High	206(64.58)	113(35.42)	319	2.778	0.249
Middle	173(64.79)	94(35.21)	267		
Low	9(90.00)	1(10.00)	10		
Tumor location					
Up	105(68.18)	49(31.82)	154	2.534	0.282
Middle	179(63.03)	105(36.97)	284		
Down	9(81.82)	2(18.18)	11		
Invasion depth					
Mucous layer	19(82.61)	4(17.39)	23	4.588	0.101
Muscle layer	101(68.24)	47(31.76)	148		
Whole layer	267(62.97)	157(37.03)	424		

### miR-378d inhibition promoted chemo-resistance, monoclonal formation, and stemness of ESCC cells

**Fig. 2:**
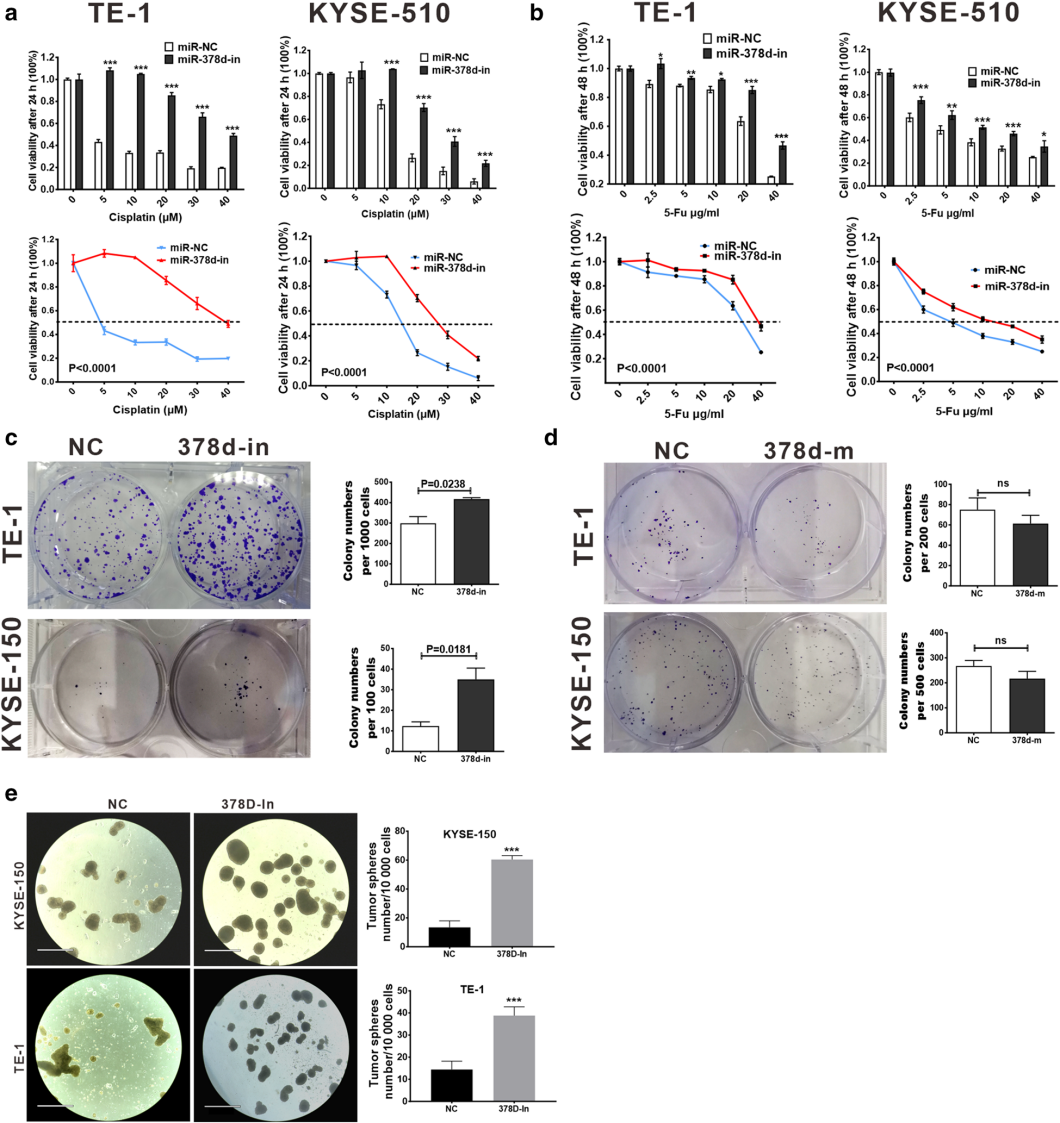
miR-378d loss expression promotes 5-Fu and cisplatin resistance, monoclonal formation, and tumor sphere formation of ESCC cells. **a**, **b** Cell viability assay; the cells were treated with different concentrations of 5-Fu or cisplatin for 24 h and then treated with CCK8 for 30 min; the OD was detected at 450 nm. *T* test and ANOVA test were used for statistical differences analysis; five repeat wells were used per concentration, *P < 0.05, ** *P* < 0.01, ***P < 0.001. **c** Silencing of miR-378d promoted the monoclonal formation of TE-1 (*P* = 0.0238) and KYSE-150 *(P* = 0.0181) cells; each experiment was repeated thrice. **d** miR-378d-mimic transient transfection detected the colony formation; each experiment was repeated thrice. **e** Tumor sphere formation assays in KYSE-150 and TE-1 cells. The graph is the summarized data of three independent experiments, ***P < 0.001. Scale bar = 890 μm

The lentivirus was used to infect ESCC cell lines to achieve the stable expression miR-378d inhibitor. Cisplatin and 5-Fu are also first-line clinical chemotherapy drugs. Our data revealed that miR-378d silencing enhanced the resistance of TE-1 and KYSE-510 cells to cisplatin at 5, 10, 20, 30, and 40 μM and significantly increased the IC50 of these cells (5–40 and 15–25 μM, respectively; Fig. 2a). Consistent with cisplatin treatment, miR-378d silencing also promoted 5-Fu resistance at 2.5, 5, 10, 20, and 10 μg/mL and increased the IC50 for TE-1 (25–40 μg/mL) and KYSE-510 (5–15 μg/mL) (Fig. 2b). miR-378d silencing promoted the monoclonal formation of TE-1 (*P* = 0.0238) and KYSE-150 (*P* = 0.0181) cells compared with control cells (Fig. 2c). The miR-378d-mimics’ transient transfection did not significantly reduce the number of colony formed but significantly reduced the clone size (Fig. 2d). Tumor sphere formation assay was performed to analyze the stem cell capability of KYSE-150 and TE-1 cells after miR-378d inhibition, and the data showed that compared with the control group, miR-378d inhibition significantly promoted sphere formation in KYSE-150 and TE-1 cells (Fig. 2e). These results suggested that miR-378d inhibition promoted chemo-resistance, monoclonal formation, and stemness.

### miR-378d inhibition promoted EMT, migration, and invasion of ESCC cells

**Fig. 3:**
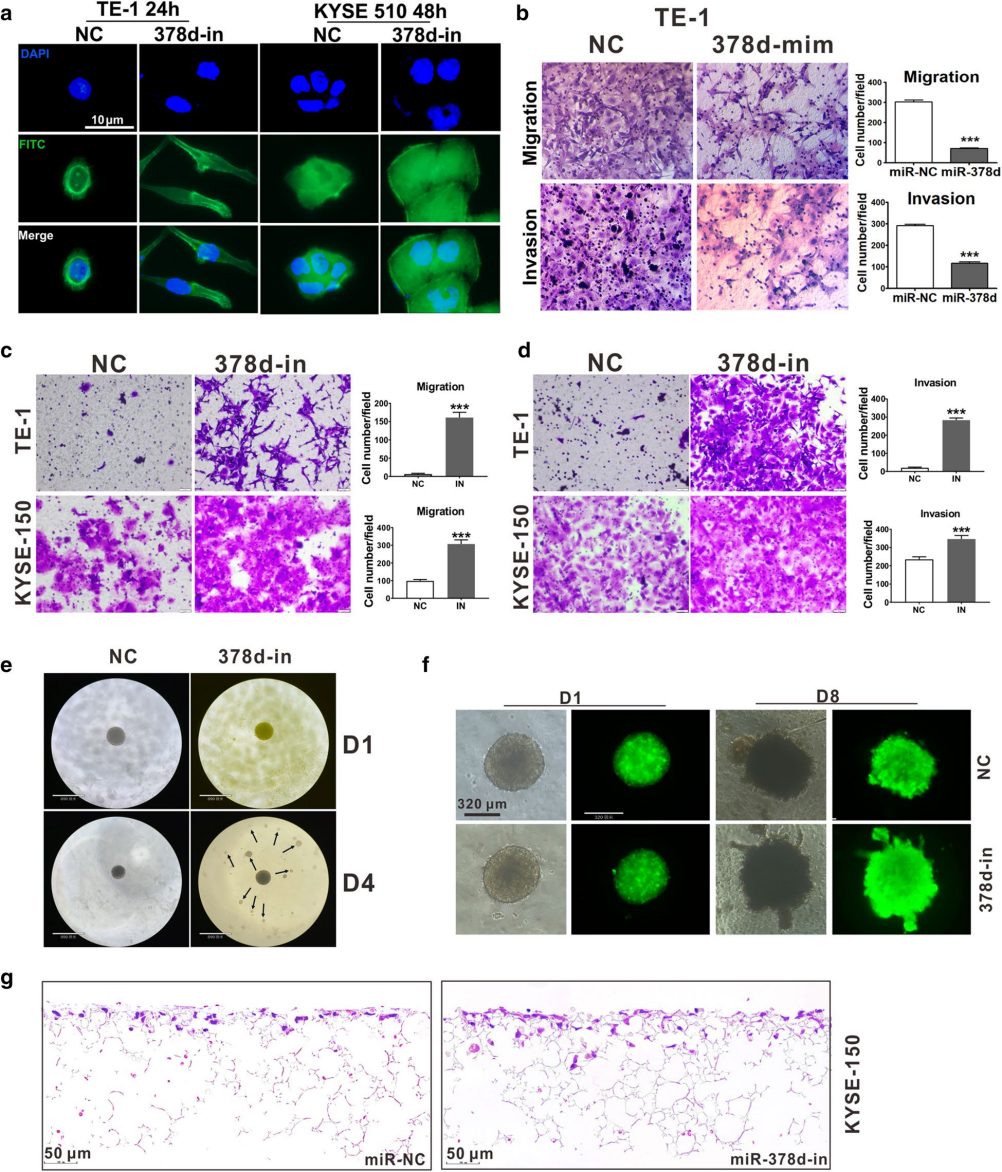
miR-378d participates in EMT, migration, and invasion of ESCC cells. **a** Phalloidin-FITC labeling the F-actin of TE-1-miR-NC (20 nM), TE-1-miR-378d-in (20 nM), KYSE150-miR-NC (20 nM), and KYSE150-miR-378d-in (20 nM) cells. **b**–**d**, Transwell assay was performed to detect the cell migration and invasion ability of ESCC cells with high or low expression of miR-378d. Random selection of five fields, ***P < 0.001. **e** 3D tumor sphere formation, suspension. **f** 3D tumor sphere formation, in Matrigel. **g** HE staining for KYSE-150 cells cultured on 3D Alvetex®, a highly loose crosslinked polystyrene scaffold, to detect the depth of cell invasion

We showed that miR-78d inhibition (20 nM) promoted microfilament skeleton formation and extension in TE-1 and KYSE-510 cells (Fig. 3a), which indicated epithelial–mesenchymal transition (EMT) occurrence. Transient transfection of miR-378d-mimics (20 nM) inhibited the migration (*P* < 0.001) and invasion (*P* < 0.001) ability of TE-1 cells (Fig. 3b), whereas miR-378d inhibition promoted the migration (*P* < 0.001) (Fig. 3c) and invasion (*P* < 0.001) (Fig. 3d) ability of TE-1 and KYSE-510 cells. KYSE-150 cells easily formed spheres in suspension state within 24 h, and miR-37d inhibition promoted the spheres’ dissemination after 48 h, whereas the control group was still forming spheres (Fig. 3e). The spheres were transferred into the Matrigel and cultured for 8 days; miR-378d inhibition showed invasive growth (Fig. 3f). The invasive ability was further detected by 3D culture on 3D Alvetex^®^, which is a highly loose crosslinked polystyrene scaffold. The data showed that miR-378d inhibition promoted cell invasion in the 3D scaffold (Fig. 3g). These results suggested that miR-378d inhibition promoted EMT, migration, and invasion of ESCC cells.

### miR-378d loss expression promotes metastasis in vivo

**Fig. 4:**
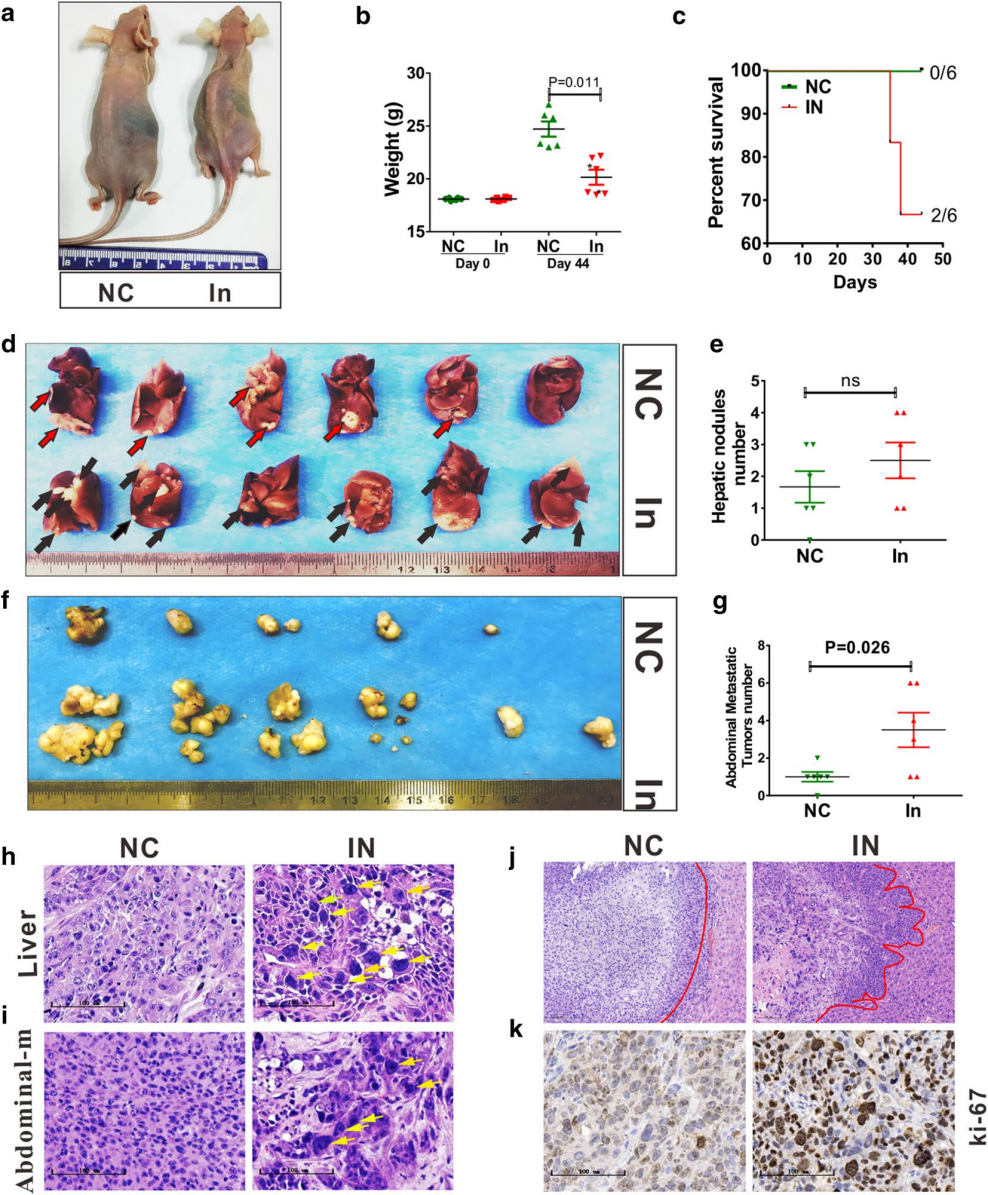
miR-378d inhibition promotes metastasis. **a** Tumor-bearing mice with liver-transplantation tumors; six BALB/c nude mice were in each group. Red and black arrows represent tumor nodules. **b** Mouse body weight at days 0 and 44, *; mouse died before day 44. Compared with NC-mice, In-mice lost body weight. *P* = 0.011. **c** Survival rate of mice. **d** Liver with transplantation tumors. **e** Number of hepatic nodules, ns: no significance. **f** Abdominal metastatic tumors. **g** Number of abdominal metastasis tumors. *P* = 0.026. **H** Orthotopic liver transplantation tumor model of nude mice used KYSE-150-miR-NC and KYSE-150-miR-378d-in cells, **h**–**j** Hematoxylin and eosin staining for liver tumors and abdominal metastatic tumors, Yellow arrows: large nuclei, Red lines: tumor margins. scale bar = 100 μm. **k** immunohistochemical staining of Ki-67 for abdominal metastatic tumors. scale bar = 100 μm

Liver is a common metastatic organ of ESCC. Thus, a liver transplantation assay was performed to analyze the metastatic ability of tumor cells without miR-378d expression. KYSE-150-miR-NC and KYSE-150-miR-378d-inhibitor cells were transplanted into the subcapsular liver of BALB/c nude mice (hereafter denoted as NC-mice and In-mice, respectively). The experiment was terminated after 44 days. The body weight of In-mice was significantly lower than that of NC-mice at day 44 (*P* = 0.011, Fig. 4a and b). Before the end of the experiment, NC-mice did not die (0/6), but two IN-mice (2/6) died at days 35 and 38 (Fig. 4c). Although the number of liver nodules in In-mice was more than that in NC-mice, no statistical difference was observed (Fig. 4d and e). Tumor cells basically metastasized to the abdominal cavity, and no metastasis was found in all lung tissues. Abdominal metastatic tumors appeared in 5/6 of the NC-mice and 6/6 of the In-mice (Fig. 4f). The number of abdominal metastatic tumors in In-mice was more than that in NC-mice (Fig. 4g, P = 0.026), and the volume was also larger in In-mice.

Although no statistical difference was found in the number of hepatic metastases, both liver tumor (Fig. 4h) and abdominal metastatic tumor (Fig. 4i) showed many large cells and significant heterogeneity after miR-378d silencing. The inhibition of miR-378d also led to invasive growth (Fig. 4j), and high expression of the proliferating marker Ki-67 (Fig. 4k). These data showed that the loss of miR-378d expression promoted tumor metastasis.

### miR378d regulated AKT1-β-catenin signaling

**Fig. 5:**
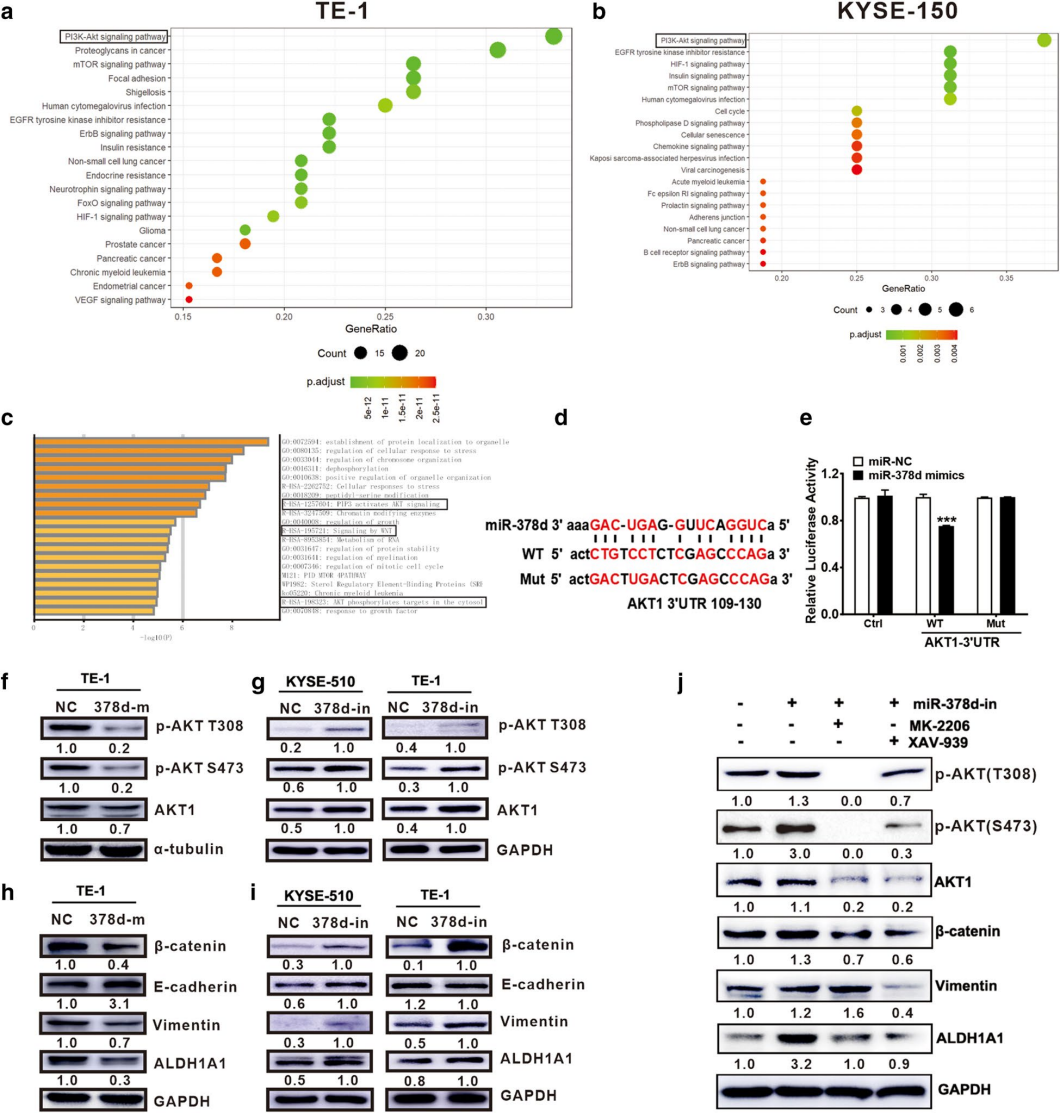
miR378d regulated AKT. **a**, **b** Differentially expressed proteins enrichment analysis. **c** Gene enrichment analysis of miR-378d predicting target genes. **d** Luciferase reporter vector design contained wild-type or mutation-type AKT1 mRNA 3′UTR (109–130 bp). **e** Dual-luciferase activity assay; miR-378d (20 nM) reduced the luciferase activity of WT-AKT1-3′UTR but not that of Mut-AKT1-3′UTR. **f**, **g** Western blot analysis detected the protein expression levels of p-AKT and AKT1; GAPDH served as an internal reference. **h**, **i** Western blot assay detected the expression levels of β-catenin, E-cadherin, ALDH1A1, and Vimentin. GAPDH served as an internal reference. **j** Western blot assay detected the protein expression levels of AKT, β-catenin, ALDH1A1, and Vimentin after miR-378d inhibition and MK2206 or XAV939 treatment in ESCC cells

Changes in cancer signaling-associated protein levels and their activation status (phospho-signaling) can be detected by Reverse Phase Protein Array (RPPA), and the pathway-enrichment statistical scatterplot showed that TE-1 NC and TE-1-PTX differential miRNA target molecules were enriched in the PI3K-AKT signaling pathways (Fig. 5a and b) both in KYSE-150 and TE-1 cells. miR-378d target genes were predicted by online software miR-TarBase (http://mirtarbase.cuhk.edu.cn/php/index.php) and TarBase V.8 (http://carolina.imis.athena-innovation.gr/diana_tools/web/index.php?r=tarbasev8%2Findex). The pathway-enrichment statistical scatterplot showed that the miR-378d target genes were enriched in AKT and WNT signaling pathways (Fig. 5c).

The miRTarBase online software predicted that AKT1 was a potential target gene of miR-378d (Fig. 5d). Dual-luciferase activity assay revealed that miR-378d targeted the 3′UTR of AKT1 mRNA (Fig. 5e). Transient transfection of miR-378d mimics to TE-1 cells resulted in the decrease of the AKT1 and p-AKT (T308 and S473) protein expression levels (Fig. 5f). Stable transfection of miR-378d inhibitor to TE-1 and KYSE-510 cells increased the total AKT1 and p-AKT (T308 and S473) expressions (Fig. 4g). The miR-378d directly regulated AKT1 expression.

The Wnt/β-catenin oncogenic signature was the AKT regulated pathway. The protein expression levels of β-catenin and the downstream target genes vimentin and ALDH1A1 decreased in cells with a high expression of miR-378d (Fig. 5h), whereas the absence of miR-378d increased the expression levels of these proteins (Fig. 5I). The EMT marker E-cadherin was significantly upregulated in TE-1 cells with high miR-378d expression and was downregulated in TE-1 cells in which miR-378 was inhibited (Fig. 5h and i). The data showed that the absence of miR-378d activated the β-catenin pathway and promoted the EMT of ESCC cells.

We also changed the AKT levels by using inhibitor MK2206 or agonist Sc79 to analyze whether the biological change was the same as that in miR-378 knockdown or overexpression. Our data showed that AKT activation promoted the migration ability (Additional file 2: Fig. S2a) but not invasion ability (Additional file 2: Fig. S2b) compared with the control cells. AKT inhibition suppressed the migration ability (Additional file 2: Fig. S2c) and invasion ability (Additional file 2: Fig. S2d) compared with control cells. With the alteration of AKT levels, the biology was largely consistent with that observed in miR-378 knockdown or overexpression.

To determine whether miR-378d regulated the expression of downstream genes (β-catenin, vimentin, and ALDH1A1) by targeting AKT1, we treated TE-1 cells with inhibitors to suppress AKT or β-catenin expression. AKT inhibitor MK-2206 downregulated the protein expression levels of AKT1, p-AKT, and the downstream β-catenin and ALDH1A1 in TE-1-miR-378d-inhibitor cells (Fig. 5j), suggesting that miR-378d silencing activated the AKT-β-catenin signaling pathway. Our data showed that inhibiting β-catenin significantly inhibited the expression of its target genes (Vimentin and ALDH1A1) and in turn inhibited AKT1 expression. The main inhibition was observed in S473 of AKT, but little effect was observed on T308 of AKT (Fig. 5j). Thus, AKT and β-catenin formed a positive feedback loop, which may play an important role in promoting malignant tumor phenotypes.

## Discussion

Most research works primarily focused on miR-378a and miR-378b [22], and only a few studies have been performed on miR-378d and inflammation [23] or infection [24]. miR-378d significantly decreased in PTX-resistant cells, but the role of miR-378d in ESCC remains unclear. In this study, we found that miR-378d inhibition promoted chemo-resistance, monoclonal formation, EMT, migration, invasion, stemness, and metastasis of ESCC cells. These results indicate that miR-378d is a tumor suppressor, and miR-378d expression is a good prognostic factor for patients with ESCC.

PTX is a mitotic inhibitor widely used in the treatment of cancer patients; it works by inhibiting cell division and inducing apoptosis. PTX treatment at high dose can lead to massive cell death, but a certain percentage of the surviving cells form PGCC, a more malignant cell type [4, 5, 25]. PGCCs exhibit higher plasticity than traditional cancer stem cells and can differentiate into a variety of tissues, including adipose tissue, cartilage, bone, and stromal or fibroblast cells [25]. PGCCs have two asymmetric cell division patterns, namely, budding and rupture, which can occur alone or together. Budding usually occurs on PGCC branches, whereas PGCC cells containing multiple nuclei usually rupture and subsequently release a large number of small cells [26].

PGCCs daughter cells promoted lymph node metastasis by expressing EMT-related proteins [27]. Spectral karyotype (SKY) analysis found that PGCC-derived daughter cells obtained a new cancer genome and a new chromosomal recombination; deletion and translocation occurred in daughter cells [28]. These studies suggested that the PGCC daughter cells have stronger migration and invasion ability than diploid tumor cells.

Our study showed that miR-378d inhibition promoted the malignant ability of ESCC cells and increased the proportion of PGCCs in the hepatic in-situ metastatic tumor model, which may promote malignant phenotype and tumor heterogeneity.

PI3K/AKT pathway are closely related to tumorigenesis, proliferation, growth, EMT, invasion, metastasis, stem-like phenotype, and drug resistance of cancer cells, but the use of PI3K or AKT inhibitors as monotherapy for different cancers is limited [29]. In this study, miR-378d directly targeted downregulated AKT1. The miR-378d inhibition activated the AKT-β-catenin signaling pathway and promoted the EMT marker vimentin and CSC marker ALDH1A1 expression, which may promote malignant phenotypes of ESCC cells.

In conclusion, miR-378d inhibition promoted chemo-resistance, monoclonal formation, EMT, migration, invasion, stemness, and metastasis of ESCC cells. miR-378d can target downregulated AKT1 and inactivate the AKT-β-catenin signaling pathway. Therefore, miR-378d expression is a good prognostic factor of patients with ESCC and regulates malignant phenotype of tumor cells through AKT signaling.

## Supplementary Information

**Additional file 1:** **Fig. S1. The precursor of the miR-378d is the MIR378D2. **mRNA expression data obtained by RNAseq in different cell lines from CCLE. **a** MIR378D1 was not expressed in all cell lines. **b** MIR378D2 was highly expressed in esophagus cell lines.

**Additional file 2:** **Fig. S2. Alteration of AKT levels manifest the same biology as miR-378d knockdown or overexpression in migration and invasion. **TE-1 cells were treated with miR-NC (20 nM), miR-378d inhibitors (20 nM), and miR-NC added AKT agonist SC79. Then, the **a **migration and **b** invasion ability were detected by transwell. TE-1 cells were treated with miR-NC (20 nM), miR-378d mimics (20 nM), and miR-NC added AKT inhibitor MK2206. Then, the **c **migration and **d** invasion ability were detected by transwell. Random selection of five fields, ****: *P*<0.0001***: *P*<0.001.

## Data Availability

The data used to support the findings of this study are available from the corresponding author upon request.
