# Supplementary figures and images for: miR-378d suppresses malignant phenotype of ESCC cells through AKT signaling
Source: Cancer Cell Int. 2021 Dec 22;21:702. doi: 10.1186/s12935-021-02403-y (PMC8697470; doi:10.1186/s12935-021-02403-y)

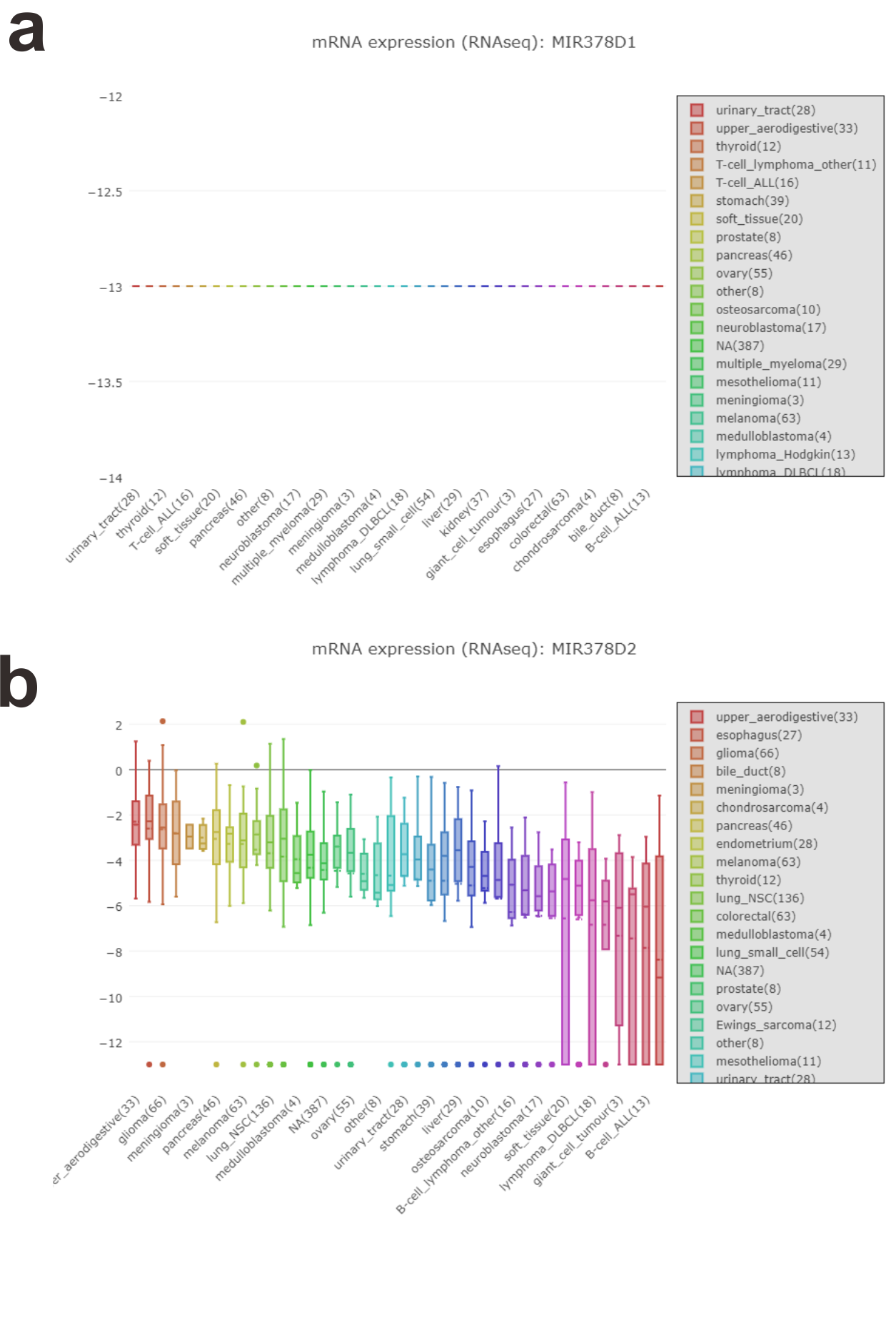

Supplement: Supplementary file 1 — Additional file 1: Fig. S1. The precursor of the miR-378d is the MIR378D2. mRNA expression data obtained by RNAseq in different cell lines from CCLE. a MIR378D1 was not expressed in all cell lines. b MIR378D2 was highly expressed in esophagus cell lines. [file 12935_2021_2403_MOESM1_ESM.tif]

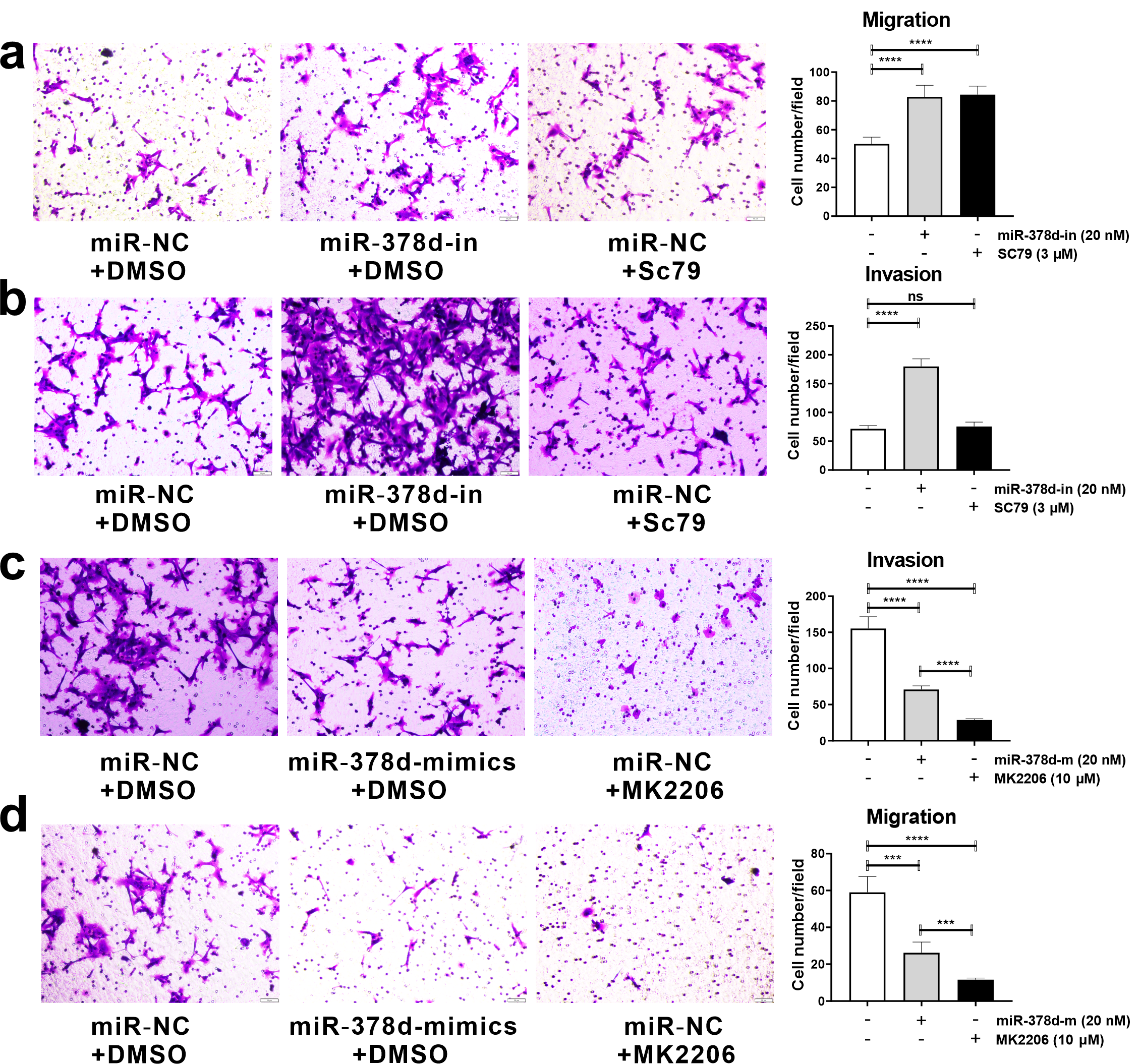

Supplement: Supplementary file 2 — Additional file 2: Fig. S2. Alteration of AKT levels manifest the same biology as miR-378d knockdown or overexpression in migration and invasion. TE-1 cells were treated with miR-NC (20 nM), miR-378d inhibitors (20 nM), and miR-NC added AKT agonist SC79. Then, the a migration and b invasion ability were detected by transwell. TE-1 cells were treated with miR-NC (20 nM), miR-378d mimics (20 nM), and miR-NC added AKT inhibitor MK2206. Then, the c migration and d invasion ability were detected by transwell. Random selection of five fields, ****: P<0.0001***: P<0.001. [file 12935_2021_2403_MOESM2_ESM.tif]
